# St. Thomas's and Bethlem Hospitals

**Published:** 1863-04

**Authors:** 


### ART. XVI.—ST. THOMAS'S AND BETHLEM HOSPITALS.

THE removal of Bethlem Hospital into a more airy and rural situation, in order that St. Thomas's may be reconstructed on its site, has of late occupied much attention both of the public and medical profession. The question thus mooted being one of special interest to psychologists, therefore demands more than a passing notice; and if our limited space permitted, we should now enter rather fully into so important an inquiry. That, however, we do not propose attempting, seeing no decisive resolution has, as yet, been arrived at by the executive authorities of the two charities just named respecting any prospective arrangement. In fact, as the whole matter in reference to change of site still remains *sub judice*, it is consequently premature to speculate what ultimate determination may be taken by the respective parties chiefly interested in the momentous point at issue, which assumes far more importance than some superficial observers may perhaps opine, who merely look at present circumstances, irrespective of future results upon persons labouring under bodily diseases or mental aberration.

Believing it may be interesting to readers not fully cognizant regarding the history of the two institutions above designated, we may mention that both were established about the same time, and are under analogous government. Bethlem Hospital, being the older, was established in Moorfields in the year 1547, and was removed to its present site fifty years ago. It is now in receipt of an annual income amounting to nearly 16,000*l.*, and gives accommodation to about 320 patients. St. Thomas's Hospital is not of such ancient date, having been founded in 1553, and in its earlier years had its struggles for existence; so much so, that the treasurer in 1564 was obliged to add to its funds by a loan of 100*l.*; and a few years afterwards half that amount was raised by pawning a lease. Subsequent endowments, however, placed this charity on a safe foundation, and its present income approaches close on 40,000*l.* per annum.

During the year 1861 there were admitted, cured, and discharged from the hospital, 46,338 patients, of which number 3935 were in-patients.

In effecting a removal of Bethlem to a country district not too distant for conveniently receiving metropolitan insane patients, and handing over the present building for St. Thomas's, much good will assuredly accrue. In the first place it will effect the preservation of that hospital in the neighbourhood in which it is most required; and the removal of the lunatics into a country situation not contaminated by smoke or immured among crowded habitations, and where open-air exercise and occupation can be had recourse to, will tend much to promote their cure. Nay, even its removal at a sacrifice of money, would ultimately be a gain to society.

Fifty years ago, when Bethlem was removed from its then horrid dungeon near London Wall, to a better and more airy site, much good resulted to the inmates; and that similarly good results will follow cannot be doubted, if the hospital was again changed from its present unfavourable situation for the cure of the insane, more especially were various modern ameliorations at the same time introduced, which have received the sanction of recent experience.

Persons taking an interest in these investigations should always remember it is a subject well worthy of notice when considering the removal of Bethlem Hospital, that its usefulness in its present situation is almost yearly becoming less extensive, on account of the want of that open-air exercise and employment deemed so necessary for curing the insane. For instance, by a reference to tables published by the institution, it will be seen that in the five years from 1828 to 1832, both inclusive, there were admitted 975 patients, and although restraint was then used, and many appliances did not exist which have been since introduced, the recoveries were 55.33 per cent., the deaths being 3.36. Whereas the total curable patients admitted during the five years ending 1860 amounted to 939; and notwithstanding the abolition of restraint, and other improvements having been introduced into the management of the hospital, the recoveries amounted only to 539 or 57·40 per cent., whilst the mortality was 5·11 per cent. This result becomes more conclusive, seeing it must be borne in mind that only those cases considered recoverable are admitted into Bethlem.

In the county asylums of England, most of which are situated in rural districts with spacious grounds, and into whose wards patients, the subjects of all forms of insanity, are admitted, the recoveries sometimes attain much higher rates than at Bethlem.

For instance, at the Gloucester asylum, in one year, they amounted to 62 per cent.; while at the French asylums, Bicêtre, for instance, the recoveries are nearly the same.

Besides the features above stated, another illustration in support of the desirability of removing Bethlem Hospital is, that latterly the admissions have become fewer in number than during various former periods. Thus, the aggregate patients admitted in the five years ending December, 1850, amounted to 1573, or 315 annually, while the admissions for the five years ending December, 1860, were only 939, or 188 annually. This indicates a great falling off, and requires some explanation, in order to account satisfactorily how so many as about one-third fewer curable lunatics were then only received into this admirably managed establishment.

An argument—indeed the only one—advanced by those who really opposed the removal of Bethlem Hospital from its present to a more rural site is, that if placed in the country it would prove often inconvenient for the friends of patients in London to visit their insane relatives. To any one conversant with the treatment of lunatics, such an assertion appears ridiculous; for, if there is one thing more than another to be avoided in the cure of demented patients, it is the frequent visits of friends, particularly during recent maniacal attacks. But it is for another absurd; since, those using that untenable argument will ascertain, on reference to the books of the hospital, that the majority of the patients are not from London. For example, during the fifteen years ending December, 1860, the number of patients admitted was, 3668, of which 1609 were from all parts of the metropolis and its neighbourhood, whilst annually 2059 were from the provinces.

Reasoning upon official data, and the statements contained in these general remarks regarding Bethlem Hospital, and further when considering impartially the fact that, modern science, directed to the better study of mental pathology and the cure of mental diseases, decisively favours the removal of lunatic patients into more salubrious and otherwise eligible situations, it is much to be hoped that in the progress of hospital reforms, Bethlem Hospital will soon find an eligible rural situation, and that no false or mistaken economy on the part of those in authority will prevent so very desirable a consummation. For the benefit of the poor and of society at large, the subject imperiously demands to be dealt with liberally. To act otherwise would indicate an extraordinary want of foresight; the whole matter is cosmopolitan, and not simply of local or individual interest, but has reference to a large class of the community should they be afflicted, whether by bodily or mental disease.

During recent discussions both at special courts of Bethlem governors, when an application regarding the contemplated purchase of that institution by the St. Thomas's authorities was considered, as likewise in prominent critical observations made in the medical or other journals, it cannot for a moment be reasonably contradicted, as was so fully shown by Dr. Webster at the late meetings of the governors of Bethlem Hospital, that a rural site, with spacious grounds, is the most proper for persons the subjects of mental diseases. There is scarcely another man in Europe who has taken more pains and incurred greater expense and loss of time than Dr. Webster to acquire a knowledge of the structure and general management of hospitals in Europe, and especially those for the insane than he has, as papers in this journal have demonstrated. His opinion and advice are therefore worthy of the greatest respect. He has already shown, as observed in recent travels, that the six public hospitals for lunatics now in course of erection in Spain, six in Sweden, and five in Denmark, are likely to be in many respects superior in arrangement to any in England, from being erected in rural districts, with such extensive grounds as will afford opportunities for employment to the patients. The opinion of Dr. Webster is that, to continue Bethlem in its present situation would become a positive injustice to the patients who seek relief within its walls, and besides must incur a great waste of money, which, if expended for the benefit of the insane in a more eligible position, could not but materially tend to their general good. It is therefore of the utmost importance that one so versed in what is necessary for proper hospital arrangement as Dr. Webster is now to be found, among the governors of Bethlem Hospital, a strenuous advocate for its removal elsewhere.

The fortunate chance which is thus offered for removing Bethlem Hospital from its present unsuited site to another, in every way more conducive for properly treating lunatics according to modern ideas in some open, airy, and rural situation, it becomes the imperative duty of the governors to accept the proposed change heartily, and on the most generous conditions. That charity will receive much more good than it bestows; at little or *no cost* to itself it will be enabled to build an hospital which can apply all modern improvements of structural kind, and Bethlem wants many such. It is admirable, none can deny, for the age when it was built and for its situation, but much more ample space in lieu of its existing confined locality and populous vicinity, will give many incalculable advantages. We have no medicines which can effectually cure a mind diseased. Opium, henbane, hyoscyamus, aconite, all the spells of the flower-world, are frequently inefficacious; they have no persistent curative powers. It is time, repose, pure air, cool and refreshing, the presence of gentle influences acting on the inscrutable nervous centre through the medium of the eye, the ear, the lungs, and so forth, which permanently restore healthy functions. This is, in many instances, the true *materia medica* of the mind, and which should never be overlooked when treating of mental maladies.

The press, the public, and medical profession are unanimous in saying that Bethlem should be vacated; but it is much more to its material interest than of St. Thomas's Hospital to seize the present opportunity to effect now for nothing, what some years hence it will be constrained to do at very great cost. Some quarter of a century ago it became known that the severe discipline, the straps, strait-waistcoats, and the fetters of that time were worse than useless, and now it is becoming known that abundant exercise, or even mere moping about, in open space, is the next great step in the curative process. Between the wards and yards of Bethlem as they now exist, and the green fields of an eligible rural site, there is as much difference as between the water of a pond and the water of a rock-spring; impure air and dirty water are alike unwholesome, but we know not the unwholesome atmosphere until it is often too late to counteract the injurious influence thereby caused on the human frame.

Among the leading articles which have lately appeared in different periodicals strenuously advocating the translation of Bethlem Hospital to a more suitable and country locality than Lambeth, so that St. Thomas's may be placed on the admirably adapted ground for a general hospital which the former institution now occupies, we would approvingly quote the conclusive and appropriate observations made thereon in a recent number of the *Lancet*:—*

"Whilst the executive authorities of St. Thomas's and Bethlehem Hospitals are investigating the money value of the latter establishment, in order that it may become the future St. Thomas's, the great benefits which a change of sites of these two institutions would confer upon humanity, whether suffering from bodily or mental disease, must not be overlooked. From the recent discussions among the governors of Bethlehem, the public begin almost to believe that some of these benevolent gentlemen are more anxious to enhance the material advantages, and hence the price, of their own structure in

* March 28th.

St. George's Fields, than to possess a new building, better adapted for lunatics, in a healthier locality, away from any urban population. Instead of haggling about the exact amount of money to be paid by one party to the other, let that question be referred to some competent umpires named by the Corporation of London—the guardian of the four Royal hospitals,—who would decide the question impartially. This point is very simple, and might be settled without difficulty, were all parties only actuated by a sincere desire to accomplish an object so likely to prove beneficial to those most concerned. The governors of each charity ought further to remember that, having been endowed by former beneficent monarchs, the one to relieve bodily disease, the other to alleviate mental alienation, even should one institution ultimately gain a few thousands by the transaction, such result will merely enable the recipient to dispense a larger share of relief.

"Should any sacrifice be requisite in order to effect such great benefits, it ought certainly to be made on the part of the Bethlehem governors, who would not only thereby get rid of a building much behind the present age in its internal capabilities for treating lunatics, but would also be able to construct another hospital in a more eligible rural situation, not far from London, which shall combine many modern improvements dictated by science and experience. In fact, it is almost impossible to calculate the advantages of the removal of Bethlehem Hospital to some more rural district, having spacious grounds adjacent for open-air exercise and for the out-door employment of patients—so necessary in the treatment of insanity.

"It is admitted on all hands that the removal of Bethlehem Hospital is urgently called for. Why does it still encumber the ground? Why are its unhappy inmates still detained from the country which invites them? At the present moment, the Paris municipality is removing the Salpétrière into the country; and every capital and large town in Europe is following, or has already adopted, analogous proceedings. Should the authorities of Bethlehem Hospital unfortunately hesitate to follow a movement so universal, and which is so cordially sanctioned by the public and by the medical profession, but finally determine to remain at Lambeth, their decision will be regarded as a deliberate adhesion to bygone theories, a perpetuation of the obstinate policy which has several times brought the administration of Bethlehem into conflict with the Government, and a wanton cruelty towards the helpless creatures committed to their charge."

The suggestion here put forward with reference to making the Corporation of London an umpire in the question at issue, is exceedingly judicious, and ought to be adopted, so as to settle all points in dispute in an amicable manner. This we may assert confidently, if the present most opportune occasion to realize two great public improvements, which will benefit numerous sufferers labouring under bodily and mental disease, be not taken advantage of, and that quickly, the time will assuredly arrive when Bethlem must emigrate to the country; and then posterity may justly assert how imprudently the authorities acted not to have effected such an important change, which during 1863 it was in their power to have accomplished by negotiations with their sister Royal hospital of St. Thomas.

---

### MEDICO-LEGAL TRIALS IN LUNACY.

